# 3D proximal tubule-on-chip model derived from kidney organoids with improved drug uptake

**DOI:** 10.1038/s41598-022-19293-3

**Published:** 2022-09-02

**Authors:** Jeffrey O. Aceves, Szilvia Heja, Kenichi Kobayashi, Sanlin S. Robinson, Tomoya Miyoshi, Takuya Matsumoto, Olivier J. M. Schäffers, Ryuji Morizane, Jennifer A. Lewis

**Affiliations:** 1 Paulson School of Engineering and Applied Sciences, Harvard University, Cambridge, MA USA; 2 Wyss Institute for Biologically Inspired Engineering, Harvard University, Boston, MA USA; 3 Nephrology Division, Massachusetts General Hospital, Boston, MA USA; 4 Department of Medicine, Harvard Medical School, Boston, MA USA; 5 Renal Division, Brigham and Women’s Hospital, Boston, MA USA

**Keywords:** Biotechnology, Stem cells, Engineering

## Abstract

Three-dimensional, organ-on-chip models that recapitulate kidney tissue are needed for drug screening and disease modeling. Here, we report a method for creating a perfusable 3D proximal tubule model composed of epithelial cells isolated from kidney organoids matured under static conditions. These organoid-derived proximal tubule epithelial cells (OPTECs) are seeded in cylindrical channels fully embedded within an extracellular matrix, where they form a confluent monolayer. A second perfusable channel is placed adjacent to each proximal tubule within these reusable multiplexed chips to mimic basolateral drug transport and uptake. Our 3D OPTEC-on-chip model exhibits significant upregulation of organic cation (OCT2) and organic anion (OAT1/3) transporters, which leads to improved drug uptake, compared to control chips based on immortalized proximal tubule epithelial cells. Hence, OPTEC tubules exhibit a higher normalized lactate dehydrogenase (LDH) release, when exposed to known nephrotoxins, cisplatin and aristolochic acid, which are diminished upon adding OCT2 and OAT1/3 transport inhibitors. Our integrated multifluidic platform paves the way for personalized kidney-on-chip models for drug screening and disease modeling.

## Introduction

Each human kidney is composed of roughly one million nephrons that filter blood and maintain electrolyte homeostasis by reabsorbing necessary nutrients back into the blood. These respective functions are achieved by glomerular and tubular subunits that reside within each nephron. The first segment of the nephron’s tubular network is known as the convoluted proximal tubule (PT). The PT is responsible for about 60–80% of nutrient reabsorption into the surrounding peritubular capillary network^1^, making it highly susceptible to damage from drugs and toxins^2^. Chronic and acute kidney injury are on the rise due to increased use of prescription drugs^3–5^. While roughly 25% of acute renal failure is drug induced^4^, predicting nephrotoxicity in preclinical in vitro human models or animal studies remains difficult. Currently, renal toxicity accounts for only 2% of failures in preclinical drug testing, yet it is responsible for nearly 20% of failures in Phase III clinical trials^3–5^. Hence, there is a critical need for patient-specific, in vitro models that more faithfully recapitulate the proximal tubule segment within human kidneys.

When cultured in two-dimensions (2D), proximal tubule epithelial cells (PTECs) typically exhibit loss of polarization and function due to limited transporter expression in the absence of physiologic cues induced by extracellular matrices and fluid flow. To overcome these limitations, considerable effort has been devoted to developing more predictive 3D models of nephrotoxicity^6–9^. Tubular networks of PTECs grown within a 3D matrigel environment form highly differentiated tubules that respond more sensitively to known nephrotoxins compared to PTECs cultured in 2D^10^. Unfortunately, these tubular networks cannot be readily perfused. To enable fluid flow, 3D microfluidic^7,11,12^ and bioprinted^13,14^ PT models have recently been introduced that exhibit enhanced polarization, function, and maturation compared to 2D culture methods. Human primary and immortalized PT cells have been used in these models; however, both cell types come with their own limitations. Primary cell lines have limited potential for self-renewal and vary considerably based on the donor^15^, while immortalized PT cell lines lack proper transporter expression compared to their *in-vivo* counterparts^16^. Hence, there is considerable interest in using renewable cell sources that are patient-specific to accurately predict nephrotoxicity in preclinical drug screening models.

Another promising approach for predicting nephrotoxicity and engineering kidney tissues is the differentiation of nephron-rich kidney organoids from human pluripotent stem cells (hPSCs)^17–23^. Kidney organoids, often referred to as “mini-organs in a dish” have been shown to elicit injury responses when exposed to known nephrotoxins^17^. Moreover, when exposed to superfusive flow, kidney organoids exhibit enhanced vascularization and maturation compared to those cultured under static conditions^18^. However, to date, no scalable method has been introduced for successfully perfusing fluid through their embedded microvasculature, glomerular compartments, and tubular segments. Understanding how flow influences drug transport and uptake within individual PT segments in kidney organoids is a crucial step towards establishing their potential for drug screening, disease modeling, and, ultimately, engineering kidney tissue for therapeutic use.

Here, we report an integrated multifluidic platform that combines organoid-derived proximal tubule epithelial cells (OPTECs) with a perfusable 3D kidney-on-chip model to enable personalized drug toxicity testing. The OPTECs are isolated from kidney organoids derived from hPSCs and seeded within our multifluidic platform composed of six individually addressable chips, each containing two co-localized channels. Each model consists of one patent lumen circumscribed by OPTECs and one empty (non-seeded) channel, embedded within a gelatin-fibrin extracellular matrix (ECM). The OPTECs form a confluent monolayer within ~ 7 days and exhibit proper apical and basal polarization, as demonstrated by acetylated alpha tubulin and LTL expression and Na^+^/K^+^ ATPase expression, basement membrane protein deposition, and basal expression of transporters in the organic cation and anion transporter families, respectively. The incorporation of two independently addressable channels per chip allows nephrotoxic drugs, cisplatin and aristolochic acid, to be introduced basolaterally, mimicking the native uptake of nephrotoxic substances in human kidneys^19^. The effect of these drugs on lactate dehydrogenase (LDH) release in the presence and absence of transport inhibitors is measured for 3D OPTECs-on-chip models and compared to control chips seeded with proximal tubule epithelial cells immortalized through stable expression of the catalytic subunit of human telomerase reverse transcriptase (PTEC-TERT1s)^20^. To our knowledge, this is the first study to report the effects of luminal flow on kidney organoid-derived proximal tubule maturation and functional response.

## Results

### 3D organoid-derived proximal tubular epithelial cells-on-chip models

**Figure 1:**
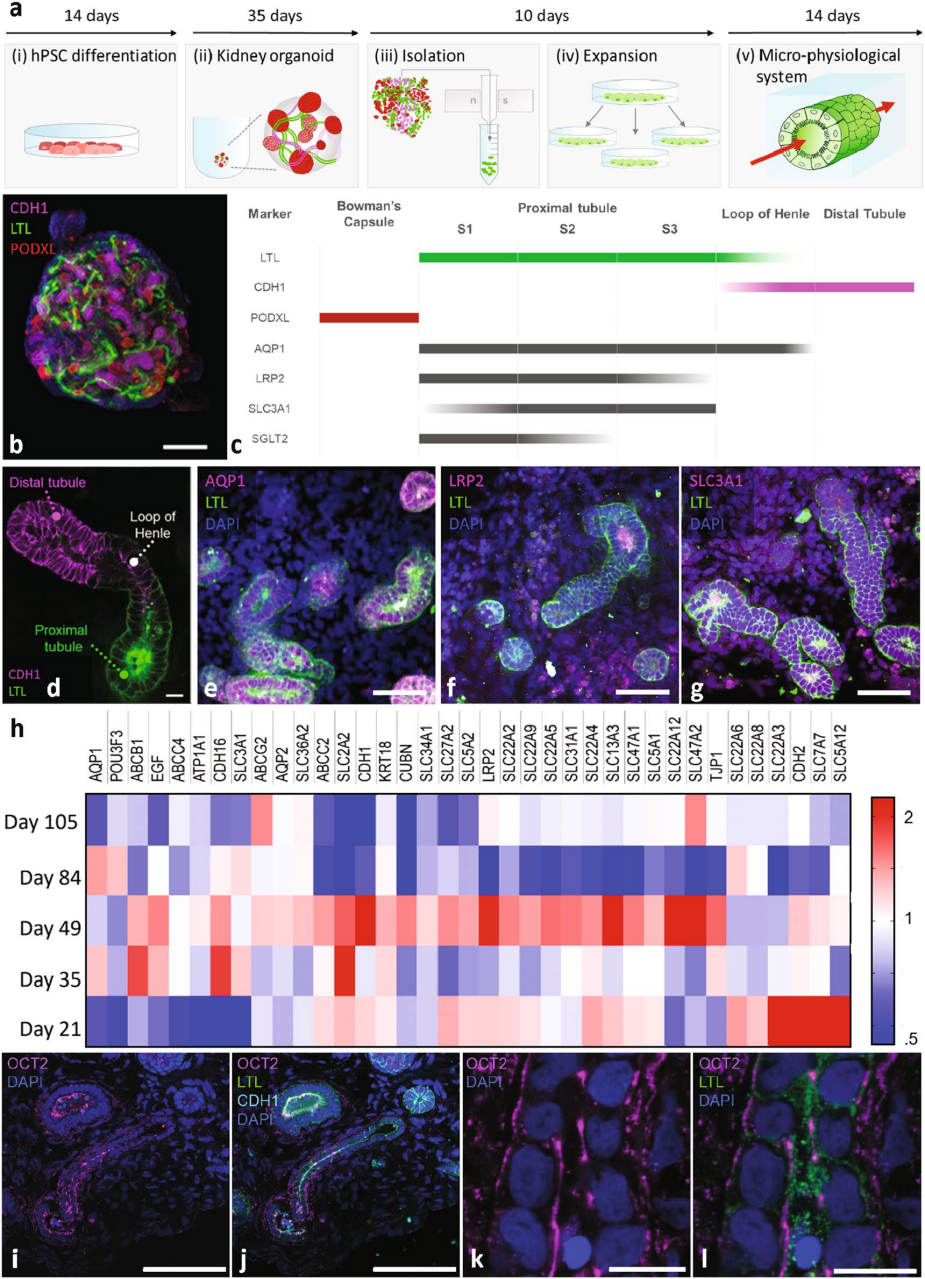
Proximal tubule epithelial cells in kidney organoids. (**a**) Schematic overview of process used to create 3D organoid-derived proximal tubule epithelial cell (3D OPTECs)-on-chip models. (**b**) Confocal image of kidney organoid (tissue-cleared) stained for PODXL^+^ (red), LTL^+^ (green), and CDH1^+^ (magenta), scale bar = 100 μm. (**c**) Schematic showing marker localization in different segments of the nephron. (**d**) Higher magnification, confocal image of kidney organoid (cryo-sectioned) stained for LTL^+^ and CDH1^+^ regions, scale bar = 10 μm. (**e**–**g**) Confocal images of kidney organoid (cryo-sectioned) stained for AQP1^+^, LRP2^+^, and SLC3A1^+^ (magenta) in nephron segments, scale bars = 50 μm. (**h**) Heat map comparing transporter expression of OPTECs isolated from kidney organoids at days 21, 35, 49, 84, and 105. (**i**, **j**) Confocal images of kidney organoid (day 49, cryos-ectioned) stained for OCT2^+^ (magenta), LTL^+^ (green), and CDH1^+^ (cyan), scale bar = 100 μm. (**k**, **l**) Higher magnification, confocal images of kidney organoid (day 49, cryo-sectioned) stained for OCT2^+^ (magenta) and LTL+ (green), scale bar = 10 μm.

Multiple steps are required to create our 3D OPTECs-on-chip models: (1) differentiate hPSCs into nephron progenitor cells, (2) produce and mature kidney organoids under static culture, (3) isolate OPTECs by magnetic-activated cell sorting (MACS)^21,22^, (4) expand these OPTECs in 2D culture, and (5) seed these OPTECs in channels embedded within a gelatin-fibrin ECM on each chip within our integrated multifluidic platform (Fig. 1a). Following the Morizane protocol^21^, we first differentiated and assembled hPSCs into multicellular aggregates composed of nephron progenitor cells (day 9 of differentiation). Upon further differentiation (days 14–49), nephron-rich kidney organoids developed that expressed glomerular (podacalyxin-like protein 1, PODXL), proximal tubule (lotus tetragonolubus lectin, LTL), loop of Henle, and distal tubule (cadherin 1, CDH1) markers (Fig. 1b, Movie S1)^21^. To identify the optimal marker for isolating OPTECs from this multicellular milieu, we evaluated LTL, aquaporin 1 (AQP1), LDL receptor related protein 2 (LRP2), solute carrier family 3 member 1 (SLC3A1), and sodium-glucose cotransport 2 (SGLT2). Since LTL is expressed in three segments of the proximal tubule, this marker allowed for greater cell yield during isolation. This marker also has less overlap with the loop of Henle, making it a better candidate compared to AQP1 (Fig. 1c–g). We used MACS sorting to isolate LTL^+^ cells, which is more benign than fluorescence-activated cell sorting (FACS) via flow cytometry.

To determine optimal time for kidney organoid maturation under static culture conditions before OPTEC isolation, we evaluated transporter expression in freshly isolated LTL^+^ cells using a Nanostring assay. A single-cell transcriptomics study carried out previously, which compared the maturity of human kidney organoids produced from two leading protocols^21,23^, showed that kidney organoids (day 26) were reflective of less mature renal tissues^24^, resembling first trimester kidneys^25^. Hence, we chose to isolate LTL^+^ cells from kidney organoids matured under static culture conditions at different time points between days 21 and 105. During this period, the average transporter expression is highest at day 49 (Fig. 1h). However, we find that the organic cation transporter 2 (OCT2) in kidney organoids (day 49) is not properly polarized to the basal side (Fig. 1i–l), as would be expected in vivo. While kidney organoids have been shown to elicit injury responses when exposed to known nephrotoxins, their tubules lack proper polarity when cultured under static conditions.

**Figure 2:**
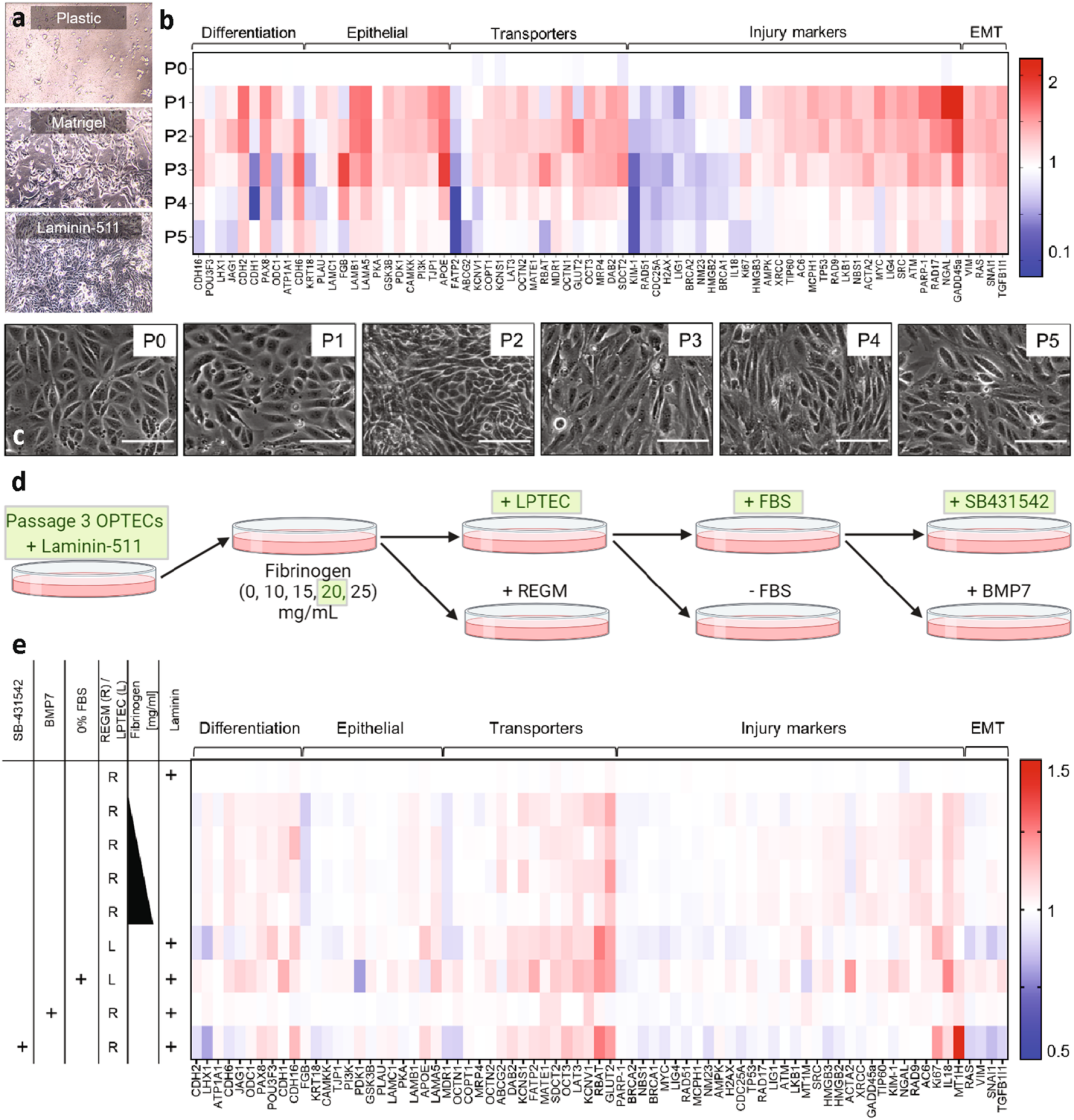
Optimizing OPTEC expansion. (**a**) Brightfield images showing OPTECs grown on plastic, matrigel, and laminin-511 substrates. (**b**) Heat map showing transporter expression of OPTECs between passages 0 (after isolation) and 5. (**c**) Brightfield images showing OPTEC populations at passages 0–5, scale bar = 20 μm. (**d**) Schematic view of the extracellular matrix (ECM), media, and media supplement conditions explored for optimizing OPTEC culture conditions. (**e**) Heat map comparing transporter expression as a function of the experimental conditions tested in (**d**).

The next step to constructing the 3D OPTEC-on-chip model is to expand the isolated LTL^+^ cells in 2D culture. OPTECs are plated on pure plastic, matrigel-coated, and different laminin-coated substrates to determine which of these substrates is most conducive to cell growth (Fig. 2a). We find that laminin-511 coated substrates yielded the fastest growth rates and resulted in OPTECs that closely mimicked the cuboidal phenotype of cultured immortalized proximal tubule cells (Supp Fig. S1). Hence, OPTECs are cultured on laminin-511 coated substrates up to five passages, where passage 0 denotes LTL^+^ cells freshly isolated from kidney organoids, to determine the optimal passage for seeding into 3D tubules. Several factors are taken into consideration, including their differentiation, epithelial, transporter, injury, and dedifferentiation markers (Fig. 2b) and cell morphology (Fig. 2c). Both the epithelial and transporter marker expression are highest for OPTECs at passage 3, which indicates that these cells should be seeded in the 3D tubules following their second passage. Brightfield images of the OPTECs between passage 0 and 5 reveal that these cells exhibit proper cuboidal morphology through passage 5. Finally, we evaluated the effects of different ECM, media, and media supplement conditions on OPTECs cultured in 2D (Fig. 2d, e). In prior work, we showed that PTEC-TERT1s grow best on an extracellular matrix composed of 25 mg/mL fibrinogen, 1wt% gelatin, 2.5 mM CaCl_2_, and 0.2wt% transglutaminase in DPBS media without calcium and magnesium^14^. We therefore focused on culturing OPTECs (passage 3) on ECMs pre-treated with laminin-511 that contained varying concentrations of 0, 10, 15, 20, and 25 mg/mL fibrinogen at a fixed gelatin content of 1wt%. We observed that the optimal transporter expression occurred for OPTECs cultured on an ECM with 20 mg/mL fibrinogen. Our optimized ECM exhibits a measured shear elastic modulus, *G*’, of ~ 4400 Pa, akin to the cortex of a healthy kidney (stiffness of ~ 4000 Pa)^13^ (Supp Fig. S2). Next, we tested the effects of two different media: (1) modified DMEM F-12 medium described by previous work^14^ (LPTEC) and (2) commercially available renal epithelial growth medium (REGM). The OPTECs exhibited higher epithelial marker expression, higher transporter expression, and lower injury marker expression when cultured in LPTEC akin to prior observations for PTEC-TERT1s^14^. To further promote cell attachment, growth, and proliferation, 1% FBS is added to the media. To minimize epithelial to mesenchymal transition (EMT), we explored the effects of transforming growth factor (TGF-β), bone morphogenic protein 7 (BMP7) and TGF-β/activin/NODAL pathway (SB431542) inhibitors (Supp Fig. S1k-l)^26^. The latter inhibitor leads to a down regulation of EMT markers as well as improved OPTEC phenotype in 2D cell monolayers, increasing the amount of time these cells could be maintained in culture. After optimizing these parameters, OPTECs with proper cuboidal phenotype grew at rates similar to those observed for PTEC-TERT1s in standard culture.

**Figure 3:**
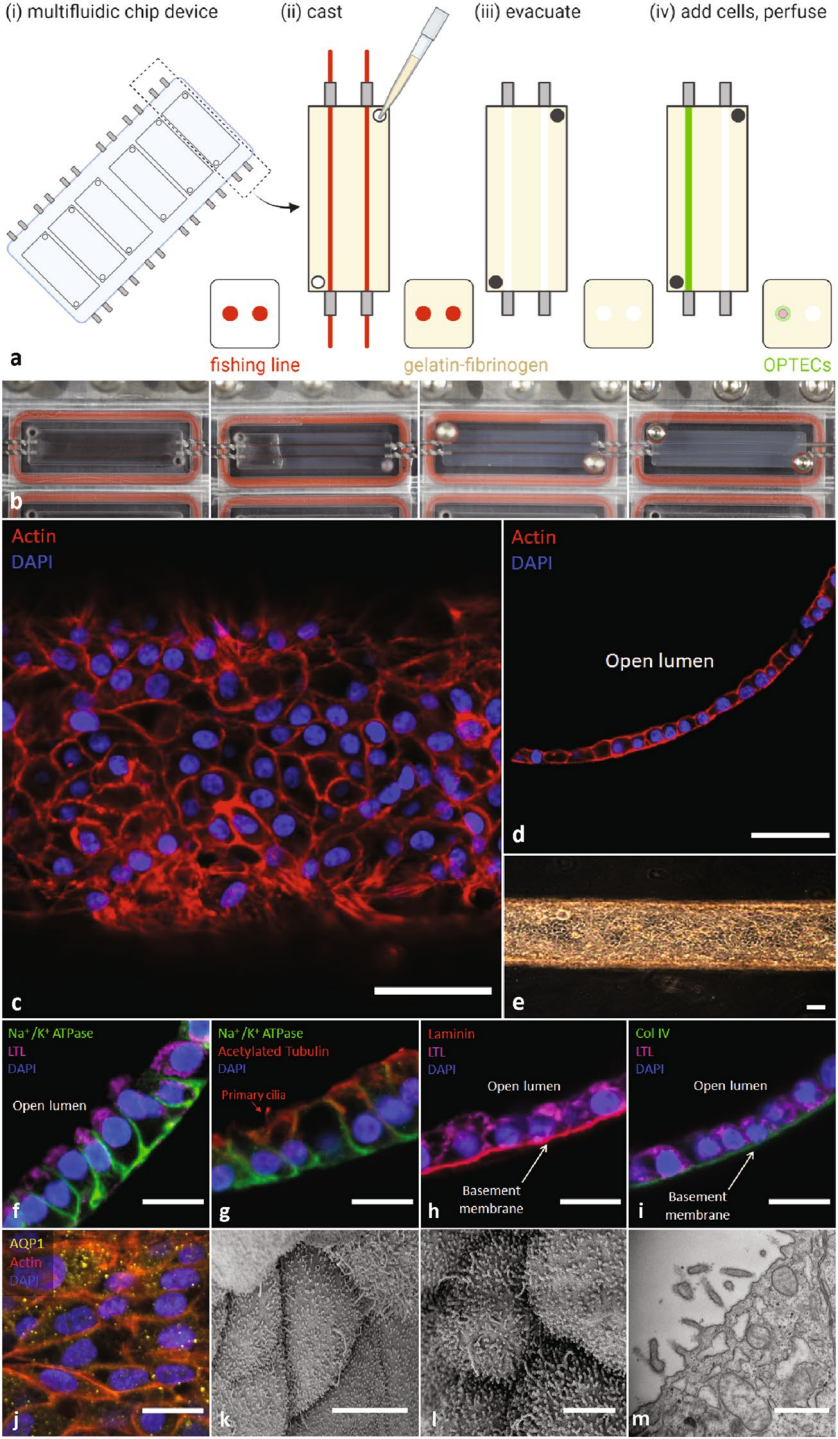
3D OPTEC-on-chip model. (**a**) Schematic views showing the processing steps used to create multiplexed, 3D OPTEC-on-chip models. (**b**) Corresponding images (left to right) of a representative chip after placing the channel templates, infilling the chip with ECM, removing the templates to create two-colocalized channels, and seeding one channel with OPTECs to create a 3D proximal tubule. (**c**) Confocal image of 3D OPTEC tubule showing actin (red) and DAPI (blue), scale bar = 50 μm. (**d**) Cross-sectional image of 3D OPTEC tubule (day 14, perfusion) highlighting the formation of a confluent monolayer, scale bar = 50 μm. (**e**) Brightfield image of 3D OPTEC tubule upon reaching confluency (day 7), scale bar = 75 μm. (**f**) OPTEC tubule (day 14, perfusion) stained for Na^+^/K^+^ ATPase (green), LTL (magenta), and DAPI (blue). (**g**) OPTEC tubules exhibit proper apical polarization of primary cilia marker, acetylated alpha tubulin (red). (**h**, **i**) Basement membrane proteins laminin (red) and Col IV (green) are deposited by OPTECs on chip. (**j**) Proper expression of AQP1 (yellow) is observed in OPTEC tubules. (**f**, **j**) scale bars = 20 μm. (**k**) SEM image highlighting primary cilia on OPTEC, scale bar = 5 μm. (**l**) SEM image of OPTEC brush border, scale bar = 20 μm. (**m**) TEM image of brush border, scale bar = 1 μm.

The final step is to create an integrated multiplexed platform composed of six, individually addressable and perfusable 3D OPTEC-on-chip models. Our original convoluted 3D proximal tubule-on-chip model was produced by multi-material bioprinting^13,14^. Since many labs lack access to this sophisticated equipment, we designed this reusable multiplexed platform using a modified pin pullout method (Supp Fig. S3). Briefly, fishing line, which serves as a channel template, is encapsulated within a gelatin-fibrinogen solution that is cast into each chip (Movie S2). Once the matrix is enzymatically cross-linked, the fishing line is removed leaving behind open tubules that can be seeded with OPTECs (Fig. 3a, b). A minimum seeding density of 10 million cells/mL is required to consistently obtain confluent tubules within a 7-day period. Importantly, we note that it is possible to create proximal tubules with diameters as small as 100 µm by this method (Supp Fig. S4). However, seeding those channels at the requisite seeding density is challenging, so we used chips with a tubule size of 380 µm in diameter. To avoid cellular aggregates, the OPTECs are filtered through a 70 µm cell strainer prior to the seeding process. To maintain a confluent epithelial layer after day 7, tubules are flushed intermittently during each media change under gravitational flow (~ 10 µL/min) for 10 min. The procedure greatly reduced the number of cellular aggregates and allowed the tubules to be cultured for several weeks post-confluency. In some instances, a small population of cellular aggregates composed of LTL^+^ and CDH1^+^ cells remain within the tubules (Supp Fig. S5), which may arise due to self-assembly. However, most tubules exhibit the desired cuboidal phenotype and assemble into a confluent epithelial monolayer in which OPTECs circumscribe the lumen (Fig. 3c, d). A combination of light microscopy, scanning electron microscopy (SEM), and transmission electron microscopy (TEM) is used to characterize OPTEC tubules after 14 days of perfusion on chip. Phase-contrast microscopy reveals that OPTECs grow throughout the tubule packing together in a columnar fashion (Fig. 3e). We confirmed the expression of the epithelial markers LTL+ and Na+/K+ ATPase and their appropriate apical and basolateral localization, respectively (Fig. 3f) by immunofluorescence (IF) imaging, akin to the in vivo phenotype. Primary cilia are also observed by staining for acetylated tubulin (Fig. 3g). The OPTECs deposit basement membrane proteins, laminin (Fig. 3h) and collagen IV (Col IV, Fig. 3i). The proximal tubule-specific water channel is also observed throughout the tubule, as revealed by the speckled pattern on their membrane surface arising from AQP1 staining (Fig. 3j). SEM images of the apical side of the OPTEC tubule reveal the formation of a confluent epithelium and the presence of primary cilia (one per cell) as well as a pronounced brush border (Fig. 3k, l). Using TEM, we obtained a higher magnification view that shows individual microvilli that protrude from the apical surface of the OPTECs as well as tight junctions between adjacent cells (Fig. 3m). Finally, we perfused FITC-inulin through the OPTEC tubules to assess their barrier function. Importantly, we find that their diffusional permeability is comparable to that observed for PTEC-TERT1 tubules (control)^13^ (Supp Fig. S6). Based on the above data, our 3D OPTEC-on-chip model appears well suited for polarized drug uptake and toxicity studies.

### Improved expression and polarization of drug transporters

**Figure 4:**
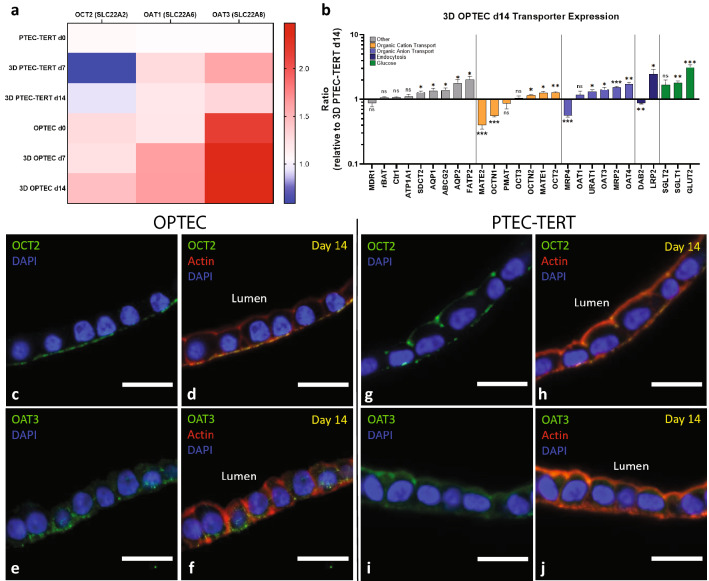
Improved transporter expression and polarization of 3D OPTECs-on-chip. (**a**) Heat map showing OCT2, OAT1, and OAT3 transporter expression for OPTECs and PTEC-TERT1s-on-chip (day 0, as seeded), after achieving confluency (day 7, perfusion), and one-week after achieving confluency on chip (day 14, perfusion). (**b**) General transporter analysis comparing OPTEC tubules normalized by PTEC-TERT1 tubules after day 14 of perfusion on chip, one sample t test, n = 6 tubules across 3 batches of OPTECs, *p < 0.05, **p < 0.01, ***p < 0.005. (**c**, **d)** Immunofluorescence images showing OCT2 (green) and DAPI (blue) staining in OPTEC tubules after day 14 of perfusion on chip. (**e–f**) Immunofluorescence images of showing localization of OAT3 (green) and DAPI (blue) in OPTEC tubules after day 14 of perfusion on chip (**g**, **h**), Immunofluorescence images showing OCT2 (green) and DAPI (blue) staining in PTEC-TERT1 tubules after day 14 of perfusion on chip, and (**i**, **j**) Immunofluorescence images of showing localization of OAT3 (green) and DAPI (blue) in PTEC-TERT1 tubules after day 14 of perfusion on chip, scale bars = 20 μm.

Known nephrotoxins, such as cisplatin and aristolochic acid, are transported basolaterally through organic cation (OCT2) and anion (OAT1/3) transporters, respectively^27,28^. To determine whether our 3D OPTEC-on-chip model exhibits proper expression and polarization of these key drug transporters, we measured the expression levels of OCT2, OAT1, and OAT3 within the OPTEC tubules over a 28-day period of perfusion using Nanostring analysis. We compare the expression of OCT2, OAT1, and OAT3 observed for OPTECs-on-chip to control chips composed of PTEC-TERT1s under the following conditions: immediately upon seeding (day 0), after the cells achieve confluency (day 7) followed by one week (day 14), two weeks (day 21), and three weeks (day 28) post confluency (Fig. 4a, Supp Fig. S7). We find that expression of each of these key transporters is higher in OPTECs compared to PTEC-TERT1s subjected to the same conditions on chip. Importantly, we observed that transporter expression is highest on day 14 for the OPTECs. A broader panel of transporters, including OCTs, OATs, endocytosis transporters, and glucose transporters is obtained for both OPTECs- and PTEC-TERT1s-on-chip after 14 days of perfusion (Fig. 4b). Interestingly, significant upregulation is observed not only for OCT2 and OAT3, but also other key PT markers including AQP1/2, SGLT2, and glucose transporter 2, GLUT2. These findings indicate that compared to immortalized cell lines, our OPTEC-based model may be more representative of in vivo PTs.

Cell polarity is essential for assessing toxicity and vectorial transport in microphysiologic analysis platforms. In kidney organoids (day 49, static), both organic cation and anion transporters are not properly polarized, as discussed above. Since OPTEC cells isolated from these organoids are used in our 3D model, we assessed whether perfusion on chip over a 14-day period enhances the polarization of key transporters, OCT2 and OAT3, by IF imaging (Fig. 4c–f). As a benchmark, we also analyzed PTEC-TERT1s perfused on chip over this same period (Fig. 4g–j). Clear basolateral polarization of OCT2 and OAT3 are only observed in OPTEC tubules. In both cases, visual expression levels of these key markers appear to also be higher in OPTEC tubules than those composed of PTEC-TERT1s, in good agreement with our Nanostring results. Taken together, the transporter expression and polarization studies reveal that OPTECs exhibit higher levels of OCT2, OAT1, and OAT3, which are localized basolaterally. Hence, our 3D OPTEC-on-chip model overcomes the lack of proper polarization in statically cultured organoids and more closely matches native proximal tubules.

### Nephrotoxic drug uptake and inhibition

**Figure 5:**
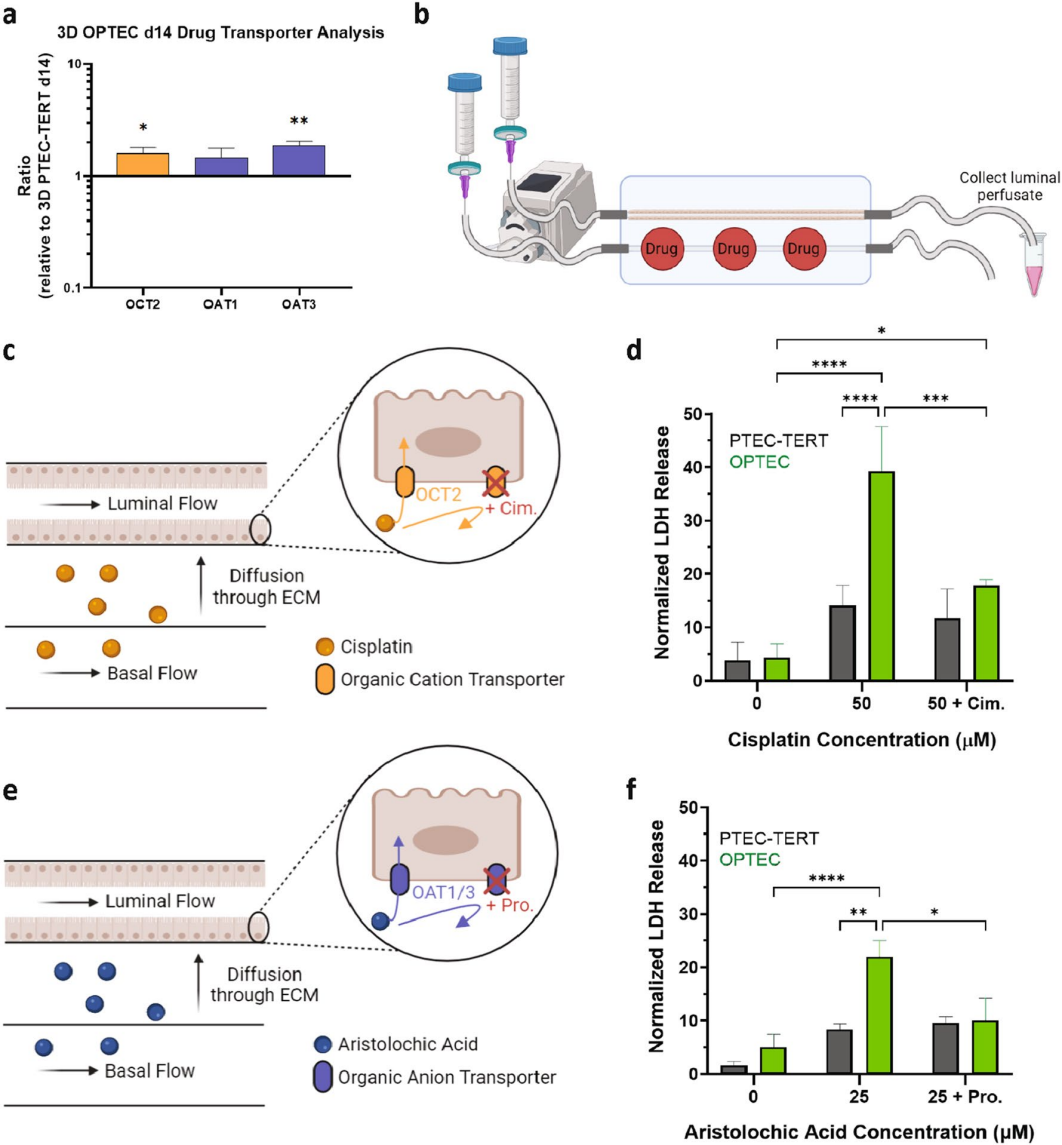
Nephrotoxicity testing. (**a**) Comparison of OCT2, OAT1, and OAT3 transporter expression in OPTEC tubules (n = 6) after day 14 of perfusion on chip compared PTEC-TERT1 tubules (controls), one sample t test. (**b**) Schematic view of experimental step used to introduce drugs and corresponding inhibitors as well as collect luminal perfusate. (**c**) Schematic view of OCT2-mediated uptake of cisplatin and inhibition using cimetidine. (**d**) Normalized LDH release observed after dosing the OPTEC (n = 4–5) and PTEC-TERT1 (n = 3–4) tubules on chip with cisplatin for 48 h, two-way ANOVA. (**e**) Schematic view of OAT1/3-mediated uptake of aristolochic acid and inhibition using probenecid. (**f**) Normalized LDH release observed after dosing the OPTEC (n = 4–10) and PTEC-TERT1 (n = 3–9) tubules on chip with aristolochic acid for 48 h, two-way ANOVA, *p < 0.05, **p < 0.01, ***p < 0.005, ****p < 0.001.

In this final set of experiments, we assessed the effects of known nephrotoxins, cisplatin and aristolochic acid, using our 3D OPTEC-on-chip model. Nanostring data showed that OPTEC tubules (day 14) expressed significantly higher levels of OCT2 and OAT3 when compared to PTEC-TERT1 tubules (day 14), as shown in Fig. 5a. To assess nephrotoxicity, luminal perfusate is collected and LDH analyses are performed. LDH is a cytosolic enzyme found in nearly all cell types, including PTECs, that is released upon cellular damage^29^. To establish a baseline level of LDH, fresh media is perfused through the OPTEC and PTEC-TERT1 (control) chips for at least 4 h. Next, samples of luminal perfusate are collected from each chip for each LDH assay. Each drug is then added to the basolateral reservoir at appropriate concentrations and allowed to perfuse for 48 h (Fig. 5b). Luminal perfusate is collected at the 48-h timepoint followed by LDH analysis. Due to differences in their baseline LDH release among different tubules across the two cell types, a normalized LDH release (LDH release at 48 h relative to baseline release) is determined for each sample, i.e., OPTEC and PTEC-TERT1 tubules without drug, with drug, and with drug and corresponding inhibitors for OCT2 and OAT1/3 transporters, respectively.

Cisplatin is an anti-tumor drug that is known to accumulate in the PT inducing nephrotoxicity. Its uptake is mediated primarily by basolateral transporter OCT2 and causes PT injury through the generation of reactive oxygen species^27^. When introduced through the basal channel on chip, cisplatin readily diffuses through the gelatin-fibrin matrix, where PT cells can uptake the drug (Fig. 5c). No differences in normalized LDH release are observed between OPTECs and PTEC-TERT1s in the absence of cisplatin. However, upon exposure to 50 µM cisplatin, OPTECs exhibited a significantly higher normalized LDH response compared to PTEC-TERT1s. Upon introduction of cimetidine (1 mM), OCT2 inhibitor^30^, the normalized LDH release is significantly decreased by more than a factor of 2 in the OPTEC tubules (Fig. 5d). By contrast, OCT2 inhibition had far less effect on the normalized LDH release observed for PTEC-TERT1 tubules exposed to the same cisplatin dose. Our observations indicate that the enhanced OCT2 expression observed for perfused OPTEC tubules leads to a physiologically relevant increase in drug uptake via this transporter.

Aristolochic acid is a potent nephrotoxin that is transported basolaterally by OAT1 and OAT3^28^. To determine the effects of aristolochic acid on our 3D models, this drug was added to the basal reservoir and allowed to diffuse through the matrix for 48 h, where PT cells can uptake the drug (Fig. 5e). In the absence of aristolochic acid, no differences are observed in normalized LDH release between OPTEC and PTEC-TERT1 tubules. When 25 µM aristolochic acid is introduced, OPTEC tubules exhibited a significantly higher LDH release compared to PTEC-TERT1 tubules. Upon adding probenecid (2 mM), OAT1/3 inhibitor^31^, the normalized LDH release is again substantially reduced for the OPTEC tubules (Fig. 5f). By contrast, OAT1/3 inhibition elicits a negligible effect on the normalized LDH release observed for PTEC-TERT1 tubules exposed to the same aristolochic acid dose. These observations further support the enhanced physiological relevance of our 3D OPTEC-on-chip model for drug screening and toxicity testing.

## Discussion

Advances in microphysiological systems have enabled the development of more physiologic proximal tubule and kidney organoid systems^11,13,14,18^. We have previously shown the functional benefits of fluidic shear stress on 3D PT models^13,14^ and kidney organoids^18^. By integrating biomanufacturing and kidney organoid development, we have now demonstrated a method for creating a perfusable, organoid-derived proximal tubule model on chip by isolating PTEC cells from kidney organoids and seeding them into 3D microenvironments that mimic native tubules. Although a longer period is required to obtain OPTECs, due to the extended organoid differentiation, cell isolation, and expansion protocols, we find that these cells exhibit the desired cuboidal morphology and rapidly assemble into a confluent epithelial monolayer when seeded in the form of 3D tubules. Upon perfusion, these OPTEC tubules exhibit proper polarization and express a broad range of proximal-tubule specific, functional markers. Importantly, they also exhibit a higher expression of basolateral drug transporters: OCT2, OAT1, and OAT3 compared to those based on immortalized PTECs. The observed decrease in transporter expression beyond day 14 may be addressed by incorporating peritubular fibroblasts into future embodiments of our model to provide additional stability^32^. We have used this 3D OPTEC-on-chip model to investigate polarized drug uptake through organic cation and anion transporters in the absence and presence of transport inhibitors. The increased transporter expression and polarization observed in our 3D OPTEC models after 14 days of perfusion directly translated to an observed increase in drug uptake and normalized LDH release by these tubules. Looking ahead, our model can be extended in two important directions by, first, producing proximal tubules with more physiologically relevant diameters (approaching 60 μm in size) and, second, by seeding the empty (basolateral) channel with endothelial cells thereby creating a vascularized OPTEC-on-chip model.

In summary, we have reported the development and characterization of a 3D organoid-derived proximal tubule-on-chip model using cells isolated from kidney organoids. Our multiplexed, perfusable OPTEC tubule model establishes a more sensitive predictor of nephrotoxicity compared to traditional models based on immortalized PTECs. Additionally, our work highlights the expected improvements that perfusion would confer on proximal tubular segments within kidney organoids subjected to physiologic luminal shear stresses. Finally, the use of PTECs obtained from hPSC-derived kidney organoids coupled with organ-on-a-chip methods opens new avenues for personalized drug screening and disease modeling.

## Methods

### Kidney organoid development and culture

Kidney organoids are prepared using the Morizane protocol^21^. In summary, hPSCs are differentiated to create SIX2^+^ nephron progenitor cells. Upon the formation of the metanephric mesenchyme (day 9 of differentiation), these cells are transferred into low attachment plates to create pre-tubular aggregates and renal vesicles. On day 14, all chemical signaling cues are removed and the kidney organoids are cultured in Advanced RPMI 1640 medium (Thermo Fisher: Gibco, 12633-012) supplemented with 1× GlutaMAX (Thermo Fisher, 35050061). The media changes are performed every 48–72 h through day 49 of static culture by replacing 95 µL of Advanced RPMI + 1× GlutaMAX from the wells.

### Isolating organoid-derived proximal tubule epithelial cells

24-well plates are prepared by coating each well with 300 µL of a 1:20 laminin-511 (Biolamina LN511) in DPBS (1× Dulbelco’s phosphate buffered saline with calcium and magnesium) solution. The plates are incubated for at least 40 min at 37 °C. MACS isolation buffer is prepared by making an ice-cold solution of 1:20 BSA MACS stock solution (Miltenyi Biotec 130-091-376) in MACS rinsing solution (Miltenyi Biotec 130-091-222). Next, d49 organoids are collected from the 96-well plates and transferred into a 15 mL conical tube. The organoids are washed twice with DPBS without calcium and magnesium and incubated in 3 mL of a 0.05% trypsin/EDTA solution for 15 min at 37 °C. The organoids are swirled in the solution every 5 min to assist in their dissociation. After 15 min, the organoids are mechanically dissociated by repeated pipetting (20 times over a 40 s period). The pipetting frequency is then increased and carried out an additional 20 times. If needed, the organoids are returned to 37 °C for 5 min in the conical tube and the pipetting steps are repeated until they are fully dissociated.

Once the organoids are fully dissociated, the trypsin/EDTA process is stopped by quenching with 9 mL of ice-cold MACS isolation buffer. Next, the samples are centrifuged in a conical tube at 240 rcf for 4 min. The supernatant is aspirated and the cells are resuspended in 1 mL of MACS isolation buffer. Any residual cell aggregates are removed by sequentially filtering this solution through 70 µm and 40 µm cell strainers. Next, the single cell suspension is centrifuged at 240 rcf for 4 min and resuspend in 80uL of biotinylated-LTL solution (1:150 B-1325 biotinylated LTL: MACS isolation buffer). The solution is placed on ice for 15 min prior to adding 1.2 mL of MACS isolation buffer and centrifuging at 240 rcf for 4 min. The supernatant is aspirated and the cells are washed with 2 mL of MACS isolation buffer prior to centrifuging again at 240 rcf for 4 min. Upon removing the supernatant, the cells are resuspended in 90 µL of MACS isolation buffer and 10µL of streptavidin magnetic beads are added to the sample, which is held on ice for 15 min. After 15 min, an additional 1200 µL of MACS isolation buffer is added and the sample is centrifuged at 240 rcf for 4 min, aspirated to remove the supernatant, and washed in 2 mL of MACS isolation buffer. The sample is centrifuged for a final time at 240 rcf for 4 min and resuspended in 500 µL of MACS isolation buffer.

MACS sorting is carried out by placing an MS column (Miltenyi Biotec 130-042-201) into the provided MACS magnet. Before adding the organoid-derived cell solution, 500 µL of MACS isolation buffer is flowed through the magnetic column into a conical tube labeled “LTL-negative”. Next, the cell solution is flowed through the magnetic column, prior to flushing the magnetic column with 500 µL of MACS isolation buffer. Another 500 µL of MACS isolation buffer is added to collect the remaining cells from the conical tube and flowed through the magnetic column. Lastly, the column is flushed with an additional 500 µL of MACS isolation buffer. The magnetic column is removed from the magnet and placed into a fresh 15 mL conical tube labeled “LTL-positive.” 1 mL of MACS isolation buffer is added to the magnetic column and manually pushed through. The LTL^+^ cells are diluted with 5 mL of pre-warmed renal epithelial cell based medium (REGM, Bioscience: Lonza CC-3191), supplemented with 1% fetal bovine serum (FBS). The sample is centrifuged at 240rcf for 4 min, aspirated to remove the supernatant and resuspended in 1 mL REGM media containing 1%FBS. The number of LTL^+^ cells are counted and seeded onto laminin pre-treated wells at a density of 70,000–100,000 cells/cm^2^. The media is changed every 24 h after isolation to remove apoptotic cells.

### Proximal tubule epithelial cell culture

Organoid-derived PTECs (OPTECs) are cultured in 24-well plates pretreated with 1:20 laminin-511 (Biolamina LN511) in DPBS with calcium and magnesium. For passages 0 and 1, the OPTECs are cultured in REGM media supplemented with 1% FBS. Upon reaching passage 2, the OPTECs are cultured using LPTEC media supplemented with 1% FBS and 10 µM SB431542 (ab120163). Each passage is performed when the cells reached ~ 90% confluency by first washing the cells with DPBS without calcium and magnesium, treating with 0.05% trypsin/EDTA for 3–5 min (or until cells lifted off the plate), followed by quenching with culture medium, centrifuging at 240 rcf for 4 min, resuspending in culture media, and seeding each laminin pre-treated well at a density of 70,000–100,000 cells/cm^2^.

For the control chips, we obtained and cultured immortalized human PTECs (PTEC-TERT1s, ATCC CRL-4031) The culture protocol provided by the manufacturer was followed with one exception. We used a modified cell media (hereby referred to as LPTEC media) composed of DMEM F-12 without glucose (pH 7.3 $\pm$ 0.05), NaHCO3 (1.2 mg/mL), D-glucose (100 mg/dL), ITS (1 × concentration, 13146-5ML; Sigma), ascorbic acid (3.5 μg/mL) triiodothyronine (5 pM), PGE1 (25 ng/mL), sodium selenite (3.65 ng/mL), hydrocortisone (25 ng/mL), and EGF (10 ng/mL).

### Extracellular matrix

An optimized extracellular matrix composed of 20 mg/mL fibrinogen, 1 wt% gelatin, 2.5 mM CaCl_2_, and 0.2 wt% transglutaminase in DPBS without calcium and magnesium is used to encapsulate the tubule and basolateral channels within each 3D OPTEC-on-chip model. The fibrinogen is made by first preparing an 80 mg/mL stock solution from lyophilized bovine blood plasma protein (Millipore). It is reconstituted in a controlled manner to prevent agitation by adding sterile DPBS without calcium and magnesium at 37 °C for between 2–3 h. Once complete, the fibrinogen solution is stored in smaller aliquots at − 20 °C for later use. A 15% (w/v) gelatin solution (Type A, 300 bloom form porcine skin, Sigma) is prepared by adding prewarmed DPBS without calcium and magnesium to the gelatin powder. This gelatin solution is then stirred for 12 h at 70 °C to allow for complete dissolving of the gelatin. Once completely dissolved, the pH is then adjusted to 7.5 by adding sufficient volume of 1 M NaOH, the gelatin solution is sterile-filtered and stored at 4 °C for later use. A 250 mM CaCl_2_ stock solution is made by dissolving CaCl_2_ pellets in sterile water and storing at 4 °C. The transglutaminase (Moo Gloo, TI) solution is made fresh for each batch of gels by dissolving the powder in DPBS without calcium and magnesium at a concentration of 60 mg/mL. This solution is held at 37 °C for 15 min for complete reconstitution and sterile filtered before use. 500 U/mL stock solutions of thrombin are created by reconstituting lyophilized thrombin (Sigma Aldrich) in sterile water and storing at − 20 °C. These aliquots are thawed within 15 min before use. After warming to 37 °C, these constituents are mixed together in the following order: DPBS without calcium and magnesium, fibrinogen, gelatin, CaCl_2_, and transglutaminase. This solution is allowed to equilibrate at 37 °C for 15 min before casting to improve the optical clarity of matrix^13^. The ECM solution is then quickly mixed with a 500 U/mL stock solution of thrombin at a ratio of 250:1 to achieve a desired thrombin of 2 U/mL in the matrix. The thrombin rapidly polymerizes fibrinogen into fibrin after the gel is cast into each chip in the MCD, as described below.

### 3D OPTEC-on-chip models

Our multiplexed devices consist of an array of six individually addressable and perfusable 3D OPTECs-on-chip models. They are fabricated following a multi-step protocol. First, we thread a channel template composed of fishing line (100–380 µm in diameter) through pins within our polycarbonate, multiplexed chip device (MCD). Unless otherwise noted, all channels are fabricated using the largest fishing line. O-rings (USA Sealing, MCS part #47417118) are then placed on each chip to seal their gel-filled compartments. The device is secured onto a metal base plate with two 50 m × 75 mm glass slides using 9 M4 12 mm screws. An enzymatically crosslinked, gelatin-fibrinogen matrix (described above) is then cast into each chip within the device. Next, small screws are used to close the holes used for the casting procedure. Finally, the entire multichip device is placed in a sterile container and allowed to cross-link at 37 °C for 0.5–2 h.

Upon sterilization, a 10 cc syringe barrel (Nordson EFD, 7012112), which serves as the media reservoir, is connected to a 0.2 µm syringe filter and 21-gauge syringe nozzle. Next, sterile two-stop Ismatec peristaltic tubing (Cole-Parmer, 95723-12) is attached to the syringe nozzle. Media is then added to the 10 cc reservoir and drawn to the end of the peristaltic tubing. After enzymatically crosslinking the gelatin-fibrin matrix but prior to connecting the tubing to the MCD, the fishing line is gently removed leaving behind two, co-localized empty channels—one of which is seeded with OPTECs to form a 3D proximal tubule and the other that remains unseeded to provide basolateral access for drug uptake studies. Once the tubing is connected to the MCD, media is flowed through the two channels to ensure all excess gel and air bubbles are removed. Lastly, an adapter composed of peristaltic tubing (2.5 cm in length) is used to connect the outlet pins to silicone tubing that returns media to 10 cc reservoirs. Clamps are used for both the inlet and outlet tubing to prevent undesired pressure changes from accumulating in the channels during media changes and handling.

Before cell seeding, the channels are coated with 40 µL of 1:20 laminin 511 in DPBS with calcium and magnesium solution at 37 °C for at least 45 min. OPTECs are used at passage 3 by first lifting the cells from the 24-well plate, then filtering the cells using a 70 µm cell strainer, resuspending at a density of 10^6^ cells/mL, then seeding 40 µL of the cell solution into the channel. PTEC-TERT1s (passage 10–20) are used as controls and resuspended at a density of 10^6^ cells/mL in LPTEC media. Both cells are seeded into the channel by removing the silicone tubing from the outlet adapter, unclamping the inlet tubing, and pipetting the cell solution into the empty channel using a p200 pipette.

Both 3D OPTEC- and PTEC-based tubules on chip are cultured using the same protocol. Before beginning flow through the cell-seeded tubules, the MCDs are placed into the incubator for 4 h. The MCDs are flipped 180° every 15 min during the first hour to ensure even coating of the tubule. After 4 h, perfusion of fresh media is initiated using a peristaltic pump at a rate of 2 µL per minute, equating to a shear stress ~ 0.1 to 0.2 dynes/cm^2^. The OPTECs typically achieve confluency by day 7, while the PTEC-TERT1s do so by day 5. Their media is changed every two days, during which a sufficient volume (5 mL per reservoir) of LPTEC media is transferred to ventilated T225s and incubated for at least 1 h to allow for proper equilibration (37 °C and 5% CO_2_). For OPTECs, 1%FBS, 1% aprotinin, 1% anti-anti, and 10 µM SB431542 is added to the media. Once the fresh media is equilibrated, the old media is removed from each reservoir. Lastly, the fresh media is added to each reservoir. To avoid cell aggregation in the OPTEC channels, these chips are flushed via gravity-driven flow at a rate of ~ 10 µL/min for 10 min to remove any debris. For PTEC TERTs, 1%FBS, 1% aprotinin, and 1% anti-anti is added to the media.

### Diffusional permeability measurements

The barrier function of the 3D OPTECs- and PTEC-TERT1s based models is assessed by measuring the diffusional permeability of 4.5 kDa inulin. This compound was selected because is neither up taken up nor secreted by PTECs in-vivo. FITC-labeled inulin (Sigma product F3272) is dissolved in prewarmed LPTEC media at a concentration of 100 µg/mL and perfused through the luminal channel at a rate of 20 µL/min for 3 min and 1.5 µL/min thereafter for 15 min. The diffusion of FITC-inulin is calculated by:

$$P_{d} = \frac{1}{{I_{1} - I_{b} }} \times \frac{{I_{2} - I_{1} }}{t} \times \frac{d}{4}$$

${P}_{d}$ is the diffusional permeability coefficient, ${I}_{1}$ is the average intensity at the initial time point, ${I}_{2}$ is the average intensity at time t, ${I}_{b}$ is the background intensity taken before perfusion of FITC-inulin, and d is the diameter of the channel^13^.

### RNA Isolation

RNeasy Mini Kit (Qiagen, 74104) was used for the RNA isolation protocol.To begin lysis of the tubules, the inlet tubing is cut, disconnected from the reservoir, and placed into a sterile 1.6 mL Eppendorf tube. Next, the outlet tubing is disconnected from the chip and 300µL of RLT buffer is pipetted through each tubule and the perfusate is collected using an Eppendorf tube. After this step, the manufacturer’s instructions are followed to isolate the RNA from the collected lysate. Briefly, 70%EtOH is added to the lysate and the solution is transferred to the spin column. A series of washing steps are carried out using the provided RW1 and RPE buffers, sequentially. Finally, the RNA is isolated using RNase-free water and immediately stored at − 80 °C.

### Nanostring gene expression analysis

Nanostring analysis is carried on samples obtained from RNA isolation at the Boston Children’s Hospital IDDRC Molecular Genetics Core Facility. First, a hybridization master mix is created by adding 70 µL nCounter Sprint hybridization buffer to a Reporter CodeSet. Next, hybridization reactions are set up by mixing 8 µL of the master mix with 5 µL of the RNA isolate, and 2 µL of the capture probe set. The reactions are then placed into a thermocycler and run overnight. The next morning, the hybridization reactions are spun down and brought to 35 µL total volume by adding 20 µL of RNase-free water. The 35 µL of sample is then loaded into a SPRINT cartridge and run using a Nanostring nCounter SPRINT profiler. The resulting data is analyzed using the nSolver4.0 software. Before comparing any sample groups, all samples are normalized to positive controls as well as housekeeping genes: ACTB, GAPDH, and TBP. Ratios comparing the sample groups are calculated in nSolver4.0 and values are plotted using GraphPad Prism 9.

### Nephrotoxicity testing

Before beginning these experiments, three important steps are carried out: (1) all reservoirs are supplied with fresh media, (2) all outlet tubing is cut to the same length, and (3) all chips are subjected to one-way rather than recirculating back to the media reservoir. After each chip within the MCDs are subjected to one-way flow (2 µL/min) for 4 h, a baseline sample of the perfusate is collected from the luminal outlet tubing for 3 h into a 1.6 mL Eppendorf tube. Next, cisplatin (50 µM) or aristolochic acid (25 µM) is delivered basolaterally on chip by manually pipetting each drug solution to the appropriate basal media reservoir. Next, all tubing is reconnected to the peristaltic pump and allowed to flow at a flow rate of 2 µL/min. After 48 h, another sample of the perfusate is collected from the luminal outlet tubing for 3 h into a 1.6 mL Eppendorf tube. All collected luminal media samples are spun down at 5000 rcf for 5 min to remove cellular debris. Each supernatant is transferred to a fresh tube and stored at − 20 °C to run the LDH assay. For samples pre-treated with inhibitors, cimetidine (1 mM) or probenecid (2 mM) is first added to the basal reservoir, delivered basolaterally, and allowed to diffuse through the gelatin-fibrin matrix during recirculating flow for 1 h.

### LDH analysis of media perfusate

LDH assays are performed according to the manufacturer’s instructions (Promega, CytoTox Non-Radioactive Cytotoxicity Assay, G1780). The frozen media samples are thawed at room temperature and used after this first thaw cycle. Next, 50 µL of each media is transferred to a 96-well plate and incubated at 37 °C for 30 min with 50 µL of CytoTox 96 reagent. 50 µL of stop solution is then added, all bubbles are removed using a syringe, and their absorbance is recorded at 490 nm using a plate reader.

Normalized LDH release is calculated using the following equation:

$$Normalized \;LDH\; release = \frac{{LDH_{48} - LDH_{baseline} }}{{LDH_{baseline} }} \times { }100$$

LDH_48_ is the recorded absorbance of the 48-h perfusate sample and LDH_baseline_ is the recorded absorbance of baseline perfusate sample before drug exposure. Normalized LDH release is calculated for each of the tubules individually and plotted into sample groups comparing OPTEC-on-chip and PTEC-TERT1-on-chip models subjected to each drug ± inhibitor.

### Imaging and immunostaining

We used an inverted Leica DM IL microscope to carry out all phase contrast microscopy (scope objectives ranging from 1.25× to 40×). Images are taken while the tubules are still in culture. For confocal microscopy, all images are taken of fixed and stained samples. To fix the chips, tubules are flushed with DPBS containing calcium and magnesium for 10 min under gravity-driven flow. Next, 10% buffered formalin is flowed through the tubular channel for 10 min. The inlet and outlet tubing are then disconnected from each chip, the MCD is removed from the baseplate, and the gels along with the embedded tubules are cut out. These samples are placed into labeled 15 mL conical tubes containing 10% buffered formalin and allowed to continue fixing for 10 min. The buffered formalin is washed away by 3 washes in DPBS with calcium and magnesium. Samples are then sliced and placed overnight in a blocking solution containing 1 wt% donkey serum and 0.125% Triton X-100 in DPBS with calcium and magnesium. The blocking solution is washed away by 3 washes of DPBS. Primary antibodies are added for 24 h at 4 °C in a staining solution containing 0.5 wt% BSA and 0.125% Triton X-100 in DPBS with calcium and magnesium. Primary antibodies are washed away by 3 washes of DPBS, with the last wash being applied overnight to remove excess, unbound primary antibody. Secondary antibodies are then incubated overnight at 4 °C in the same staining solution used for the primary antibodies. Samples are then stained with DAPI and washed for 2 h in DPBS before imaging. Antibody lists for kidney organoid and tubule staining are provided Supplementary Tables 1–2, respectively. For confocal images, an upright Zeiss LSM 710 with water-immersion scope objectives (ranging from 10 to 40×) is used with spectral lasers at 405, 488, 514, 561, and 633 nm. ImageJ software is used to reconstruct all confocal images.

### Electron microscopy

For transmission electron microscopy (TEM), OPTECs are fixed using 2.5% glutaraldehyde, 1.25% paraformaldehyde, and 0.03% picric acid in 0.1 M sodium cacodylate buffer (pH 7.4) for a minimum of several hours. Small samples (1 mm × 1 mm) are removed and washed in 0.1 M cacodylate buffer and bathed in 1% osmiumtetroxide (OsO_4_) (EMS) and 1.5% potassiumferrocyanide (KFeCN_6_) (Sigma) for 1 h, washed in water 3× and incubated in 1% aqueous uranyl acetate (EMS) for 1 h followed by 2 washes in water and subsequent dehydration in varying grades of alcohol (10 min each; 50%, 70%, 90%, 2 × 10 min 100%). The samples are then put in propyleneoxide (EMS) for 1 h and incubated overnight in a 1:1 mixture of propyleneoxide and TAAB Epon (Marivac Canada Inc. St. Laurent, Canada). The following day the samples are embedded in TAAB Epon and polymerized at 60 °C for 48 h. Ultrathin sections (about 60 nm) are cut on a Reichert Ultracut-S microtome, placed on copper grids stained with lead citrate and examined in a JEOL 1200EX Transmission electron microscope and images are recorded with an AMT 2 k CCD camera. Image analysis is performed using ImageJ software.

For scanning electron microscopy (SEM), perfused OPTECs in 3D are fixed using 10% buffered formalin for 1 h. The samples are thinly sliced (~ 1 mm thick) to expose cells circumscribing the open lumen. The fixative is washed away using PBSx2 and subsequent dehydration in varying grades of ethanol (20 min each; 30%, 50%, 70%, 90%, 3 × 20 min 100%). The samples are then placed in 50% ethanol and 50% hexamethyldisilazane (HMDS) for 30 min followed by 100% HMDS 3 × 30 min. All steps are performed in a closed and sealed glass container. After the final washing with HMDS, the samples are removed and placed in an open container under N_2_ in the fume hood to dry. Dried samples are mounted to aluminum pin mounts using conductive carbon tape, sputter coated with gold, and imaged with a Tescan Vega SEM.

## Supplementary Information

Supplementary Information 1.

Supplementary Video 1.

Supplementary Video 2.

## Data Availability

The datasets used and/or analysed during the current study available from the corresponding author on reasonable request.
