# Supplementary material for: 3D proximal tubule-on-chip model derived from kidney organoids with improved drug uptake
Source: Sci Rep. 2022 Sep 2;12:14997. doi: 10.1038/s41598-022-19293-3 (PMC9440090; doi:10.1038/s41598-022-19293-3)
Supplement: Supplementary file 1 — Supplementary Information 1. [file 41598_2022_19293_MOESM1_ESM.pdf]

3D proximal tubule-on-chip model derived from kidney organoids with improved drug uptake

Jeffrey O. Aceves[1,2], Szilvia Heja[2], Kenichi Kobayashi[3,4], Sanlin S. Robinson[1,2], Tomoya Miyoshi[4,5], Takuya Matsumoto[2,3,4,5], Olivier J.M. Schäffers[4,5], Ryuji Morizane[2,3,4,5*], and Jennifer A. Lewis[1,2, *]

[1]Paulson School of Engineering and Applied Sciences, Harvard University, Cambridge, MA, USA. [2]Wyss Institute for Biologically Inspired Engineering, Harvard University, Boston, MA, USA. [3]Nephrology Division, Massachusetts General Hospital, Boston, MA, USA
[4]Department of Medicine, Harvard Medical School, Boston, MA, USA.
[5]Renal Division, Brigham and Women's Hospital, Boston, MA, USA.
[*]Corresponding author's email: rmorizane@mgh.harvard.edu and jalewis@seas.harvard.edu

Supplementary Information

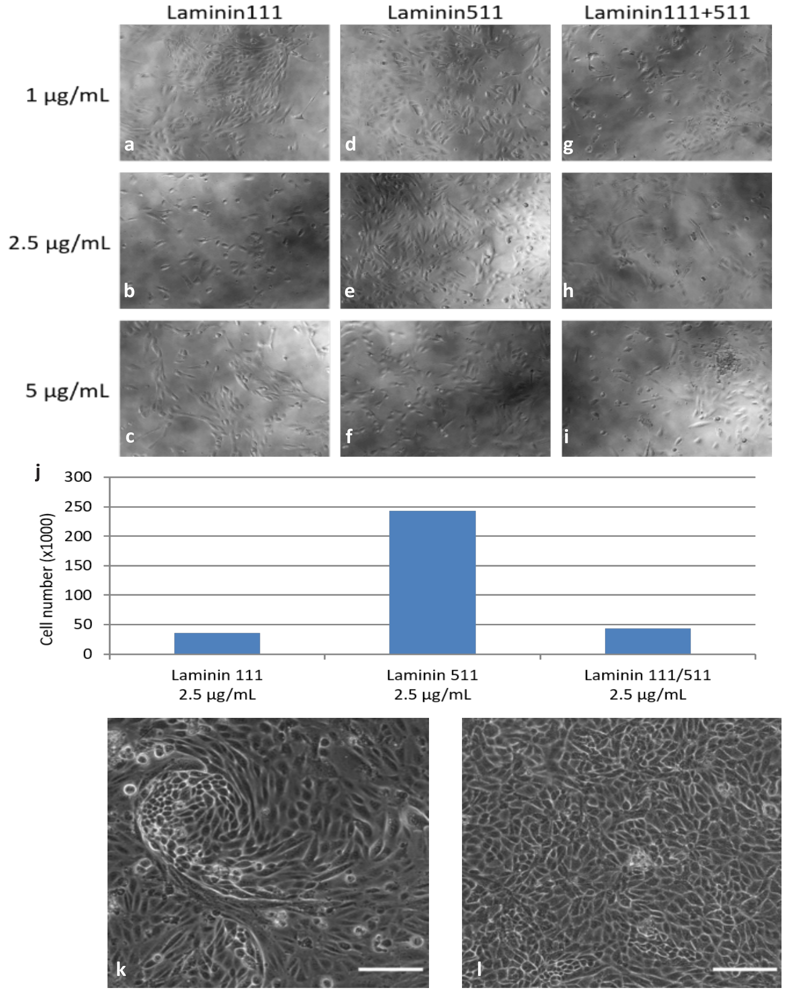

Figure S1. OPTEC culture. (a-i) 2D culture of OPTECs on laminin 111, laminin 511, and laminin 111/511-coated substrates at varying concentrations of 1 µg/mL, 2.5 µg/mL, and 5µg/mL. **(j)** Plot of number of OPTECs at day 11 when cultured on 2.5 µg/mL of laminin 111, laminin 511, or laminin 111/511. **(k)** OPTECs undergo dedifferentiation (epithelial to mesenchymal transition) of during prolonged culture. **(i)** OPTECs treated with 10 µM of TGF-β inhibitor, SB431542, exhibit cuboidal cell morphology during prolonged cell culture, scale bars = 100 µm.

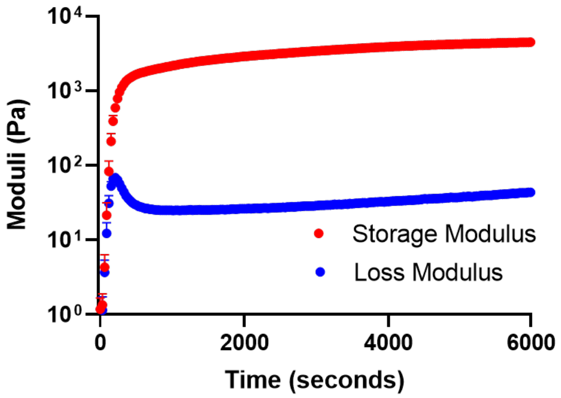

Figure S2. **Optimized matrix rheology.** Semilog plot of storage (*G*') and loss (*G*'') moduli of our optimized extracellular matrix (ECM), which is composed of 1 wt% gelatin and 20 mg fibrinogen.

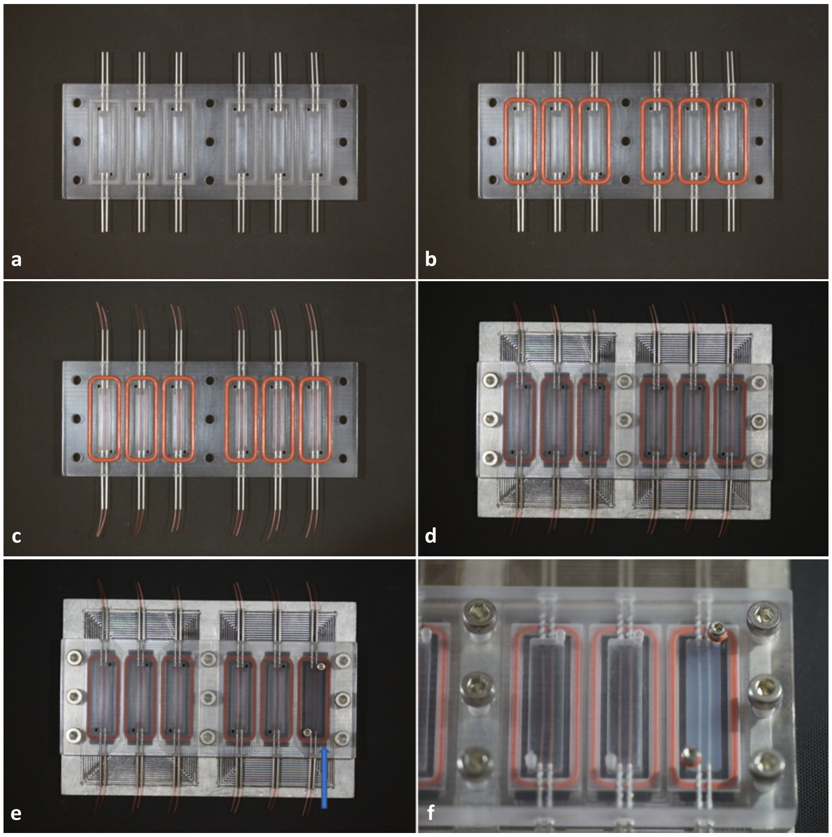

Figure S3. Multiplexed 3D proximal tubule-on-chip device fabrication. (a) Polycarbonate housing. **(b)** O-rings are placed into machined indentions within each chip to create a proper seal. **(c)** Fishing line, which serves as a channel template, is threaded through two sets of pins on each chip in the device. **(d)** Glass slides (50mm X 75mm) are placed into a steel base plate and the polycarbonate housing with O-rings and fishing line is screwed on using 12mm M4 screws. **(e)** A gelatin-fibrinogen solution is cast into each chip to fully encapsulate the templated channels. **(f)** After enzymatically cross-linking this solution, the fishing line is removed leaving behind empty channels embedded within a gelatin-fibrin matrix that can be seeded with cells.

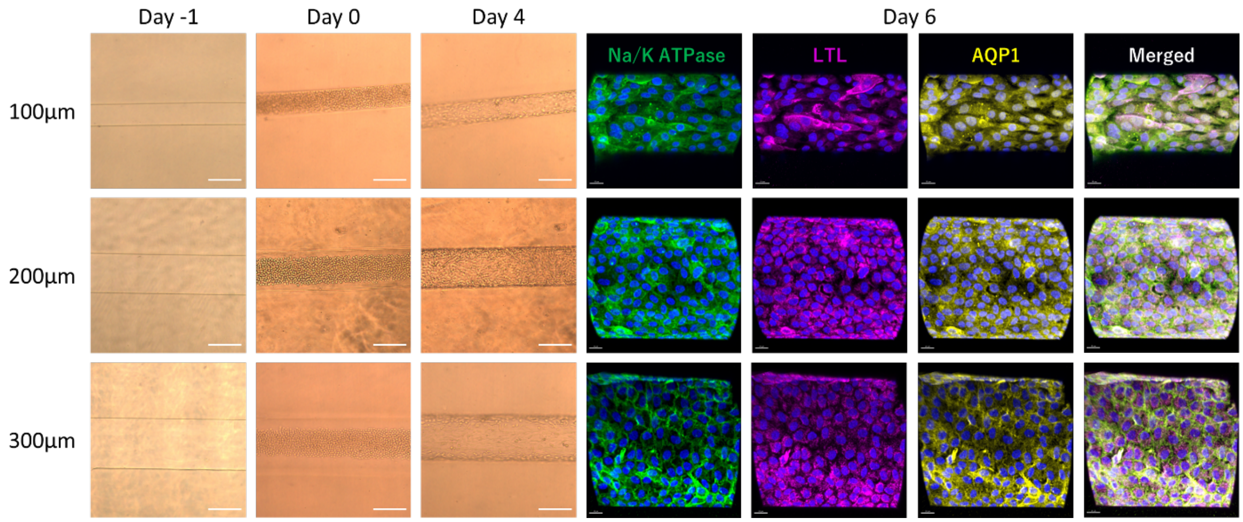

Figure S4. Proximal tubules of varying diameter in multiplexed chip devices. (Top row) Bright field and confocal images of proximal tubules produced using (100 µm diameter fishing line) in our multiplexed chip device, which shows (from left to right) empty channel (Day -1), seeding of PTEC-TERTs (Day 0), formation of confluent epithelium (Day 4), and confocal image of proximal tubules (Day 6) stained for different functional markers (Na^+/K^+ ATPase, LTL, AQP1, and merged). **(Middle row)** Corresponding image sequence for proximal tubules produced using fishing line that is 200 µm in diameter. **(Bottom row)** Corresponding image sequence for proximal tubules produced using fishing line that is 300 µm in diameter. Scale bars = 200 µm (bright-field images) and 20 µm (confocal images).

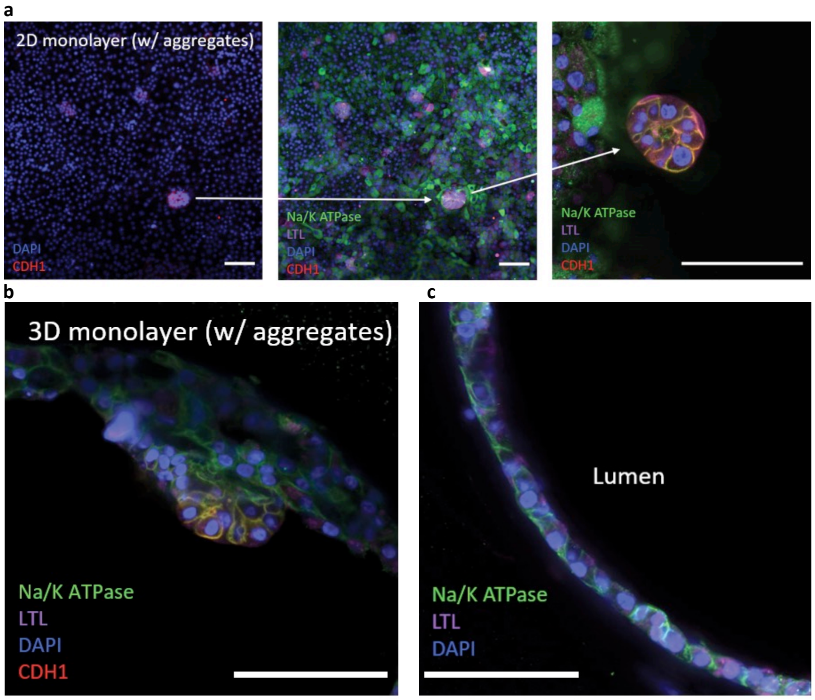

Figure S5. Multicellular aggregates. (a) Immunofluorescence image of a OPTEC monolayer in 2D culture stained for Na/K ATPase (green), LTL (magenta), CDH1 (red), and DAPI (blue). **(b)** Confocal image of OPTEC tubule-on-chip without flushing step, which reveals the presence of multicellular aggregates. In **(a-b)**, all aggregates observed express CDH1. **(c)** Confocal image of OPTEC tubule improved protocol with intermittent flushing steps that mitigate multicellular aggregate formation, all scale bars = 100 μm.

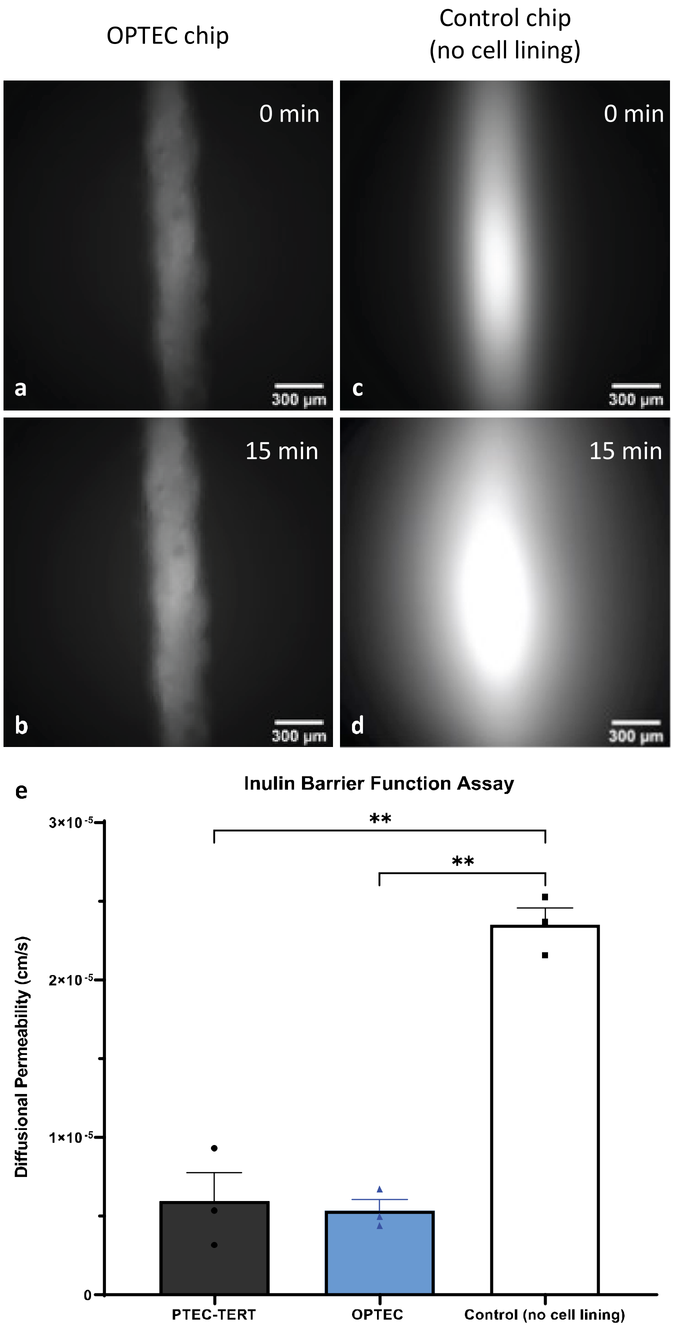

Figure S6. Diffusional permeability measurements. A 4.5kDa FITC-inulin dye in LPTEC media is perfused through luminal channels at varying times: **(a)** t=0 min and **(b)** t=15 min for confluent OPTEC tubules, and **(c)** t=0 min and **(d)** t=15 min for control channels (unseeded). **(e)** Diffusional permeability for tubules seeded with PTEC-TERT1s or OPTECs as well as the control (unseeded channel), scale bar = 300 µm, n=3, two-way ANOVA, **$p<0.01$.

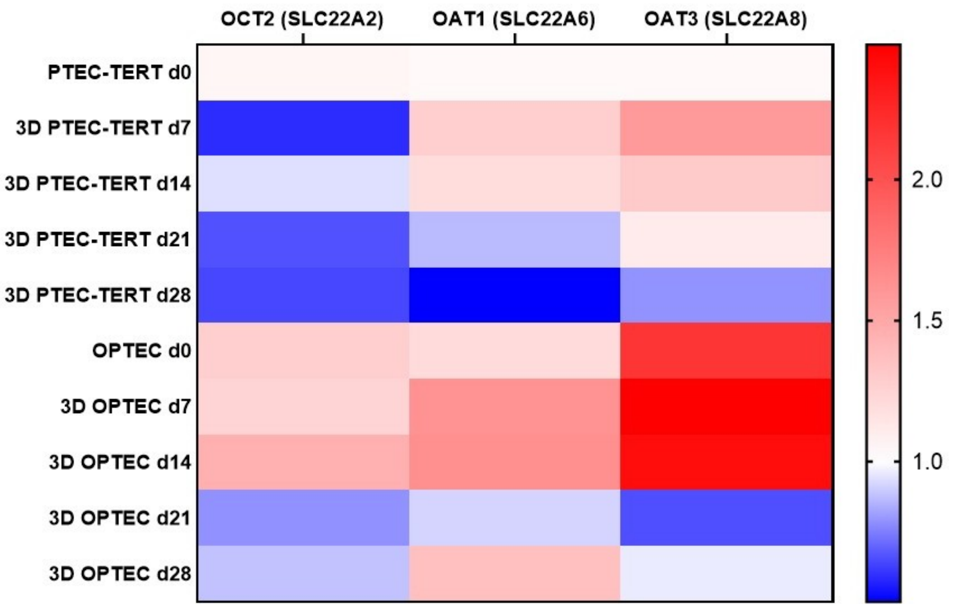

Figure S7. Drug transporter expression. Heat map showing expression of OCT2, OAT1, and OAT3 assessed via Nanostring for OPTEC and PTEC-TERT1 tubules (n=4-6) perfused on chip from day 0 through day 28. All data normalized to PTEC-TERT1 transporter expression on day 0 (immediately after seeding).

Table S1. Immunostaining reagents for organoid staining.

Antibody for organoid sections	Manufacturer	Catalog #
CDH1	Abcam	ab11512
LTL	VectorLabs	B-1325
PODXL	R&D	AF1658
AQP1	Abcam	ab2219
LRP2	Sigma Aldrich	MABS489
SLC3A1	Sigma Aldrich	HPA038360
OCT2	NSJ Bioreagents	F43298
OAT1	Biorbyt	orb11177
SGLT2	Abcam	ab85626

Table S2. Immunostaining reagents for tubule staining.

Antibody or Stain for tubules	Manufacturer	Catalog #	Host Species & Reactivity	Concentration
Na/K ATPase	Abcam	ab76020	Rabbit anti-human	1:300
LTL	Vector Lab	B-1325	N/A	1:200
OCT2	Abcam	ab170871	Rabbit anti-human	1:200
AQP1	Santa Cruz	SC25287	Mouse anti-human	1:300
Laminin	Abcam	ab11575	Rabbit anti-human	1:300
Col IV	Abcam	ab6586	Rabbit anti-human	1:300
Acetylated alpha tubulin	Abcam	ab24610	Mouse anti-human	1:300
ActinRed	Life Technologies	R37112	N/A	2 drops per mL
ActinGreen	Life Technologies	R37110	N/A	2 drops per mL

Movie S1. 3D rendering of confocal images obtained on kidney organoids under static culture (day 49) showing PODXL (red), LTL (green), CDH1 (magenta), and DAPI (blue), scale bar = 10 µm.

Movie S2. Casting gelatin-fibrinogen solution around channel templates housed within the multiplexed, perfusable 3D OPTEC-on-chip devices.
